# Type C Personality: Conceptual Refinement and Preliminary Operationalization

**DOI:** 10.3389/fpsyg.2020.552740

**Published:** 2020-09-16

**Authors:** Karolina Rymarczyk, Anna Turbacz, Włodzimierz Strus, Jan Cieciuch

**Affiliations:** ^1^Institute of Psychology, Cardinal Stefan Wyszyński University in Warsaw, Warsaw, Poland; ^2^URPP Social Networks University of Zurich, Zurich, Switzerland

**Keywords:** Type C personality, Circumplex of Personality Metatraits, submissiveness, restricted affectivity, cancer-prone personality

## Abstract

In this paper, we have presented our proposal for reconceptualization and operationalization of Type C (cancer-prone) personality. Based on theoretical analyses, taking into account both the literature on Type C and models of personality structure, we have proposed a two-facet structure of Type C, comprising *Submissiveness* (the interpersonal aspect) and *Restricted Affectivity* (the intrapersonal aspect). The study devoted to the validation of the measure of Type C involved 232 participants aged 18–70 (*M* = 29.35, *SD* = 8.93; 54% male). We used (a) our proposed measure of Type C personality and (b) the Circumplex of Personality Metatraits Questionnaire (CPM-Q-SF; Strus and Cieciuch, 2017), assessing personality metatraits. The measure of Type C proved to have acceptable internal consistency (Cronbach’s alpha was 0.85 for Submissiveness and 0.78 for Restricted Affectivity). The measurement model in confirmatory factor analysis with two latent variables proved to be well-fitted to the data. We have also confirmed the hypothesis concerning the location of the two facets of Type C personality close to each other in the theoretically predicted area between the Delta-Plus/Self-Restraint and Beta-Minus/Passiveness metatraits (in the Circumplex of Personality Metatraits). The clinical value of the theoretically refined Type C can be tested in the next step in research on patients with cancer.

## Introduction

### Type C: The Search for Psychological Determinants of Cancer

The belief that somatic diseases depend also on psychological factors has been the underlying assumption of many studies that sought to identify those personality characteristics that increased the risk of specific somatic diseases or were responsible for general susceptibility to diseases (Friedman and Rosenman, 1959; Greer and Morris, 1975; Denollet et al., 1995; Dolińska-Zygmunt, 2001b; Ogińska-Bulik and Juczyński, 2008; Horwood et al., 2015; Šmigelskas et al., 2015). One of the personality constructs claimed to be associated with the occurrence of cancer is Type C personality (Eysenck, 1994; Bozo et al., 2014; Habibi et al., 2015), also referred to as Type C behavior (Greer and Watson, 1985), Pattern C behavior (Dolińska-Zygmunt, 2001b), or cancer-prone personality (Eysenck, 1994; Watson et al., 1999).

**Figure 1:**
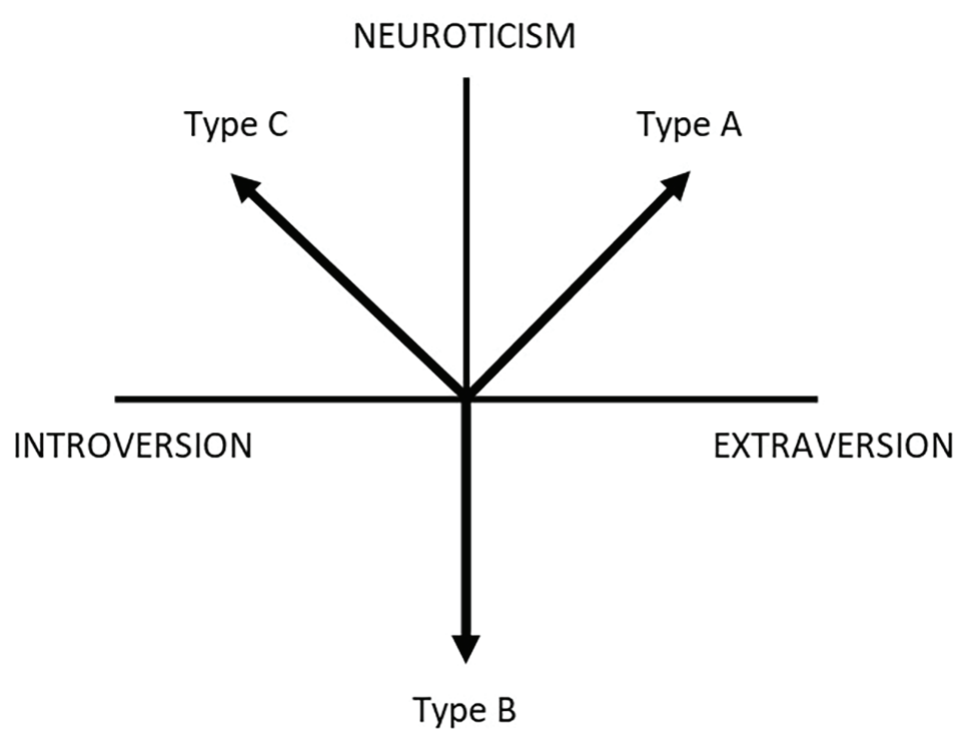
Hypothetical relations of Types A, B, and C to extraversion and neuroticism (Eysenck, 1991).

The introduction of Type C into the literature is usually attributed to Greer and Morris (1975), who conducted research on a sample of women with breast cancer and found the co-occurrence of cancer with a certain pattern of behavior associated with abnormal expression of emotions, which they later named Type C behavior (Greer and Watson, 1985). The characteristics of Type C and its relations to other personality types and traits were also the subject of the work of Eysenck (1991). What is particularly often provided in the literature is the graphic presentation of Types C (cancer-prone personality), Type A (coronary heart disease-prone personality), and Type B (normal, not disease-prone personality) in relation to the traits of extraversion and neuroticism, proposed by Eysenck (1991), as shown in Figure 1.

According to this perspective, cancer-prone personality (Type C) is associated with neuroticism and introversion, while coronary heart disease-prone personality is associated with neuroticism and extraversion (Eysenck, 1991). Empirical results, however, did not always confirm this pattern of theoretical relations, and sometimes, they even directly contradicted it. Already in the first study of Kissen and Eysenck (1962) conducted on a sample of men with lung cancer, it turned out that the occurrence of cancer was related to a low rather than high level of neuroticism. Also in his other publications, Eysenck (1985) discussed studies in which it was low neuroticism (e.g., Morris et al., 1981) or high extraversion that was significantly related to cancer incidence (e.g., Coppen and Metcalfe, 1963; Hagnell, 1966). There were attempts to explain the empirically found negative relationship between neuroticism and cancer as stemming from the emotional repression that may accompany low neuroticism (Eysenck, 1991) and the negative link between cancer and introversion as due to the better condition of the immune system in introverts (Eysenck, 1985).

A number of further studies on cancer patients revealed additional links between Type C and psychosocial factors but they were not always consistent either. For example, a study by Garssen and Goodkin (1999) confirmed that a low level of social support, a tendency toward helplessness, and repression of negative emotions were significant in the development of cancer. Lehto et al. (2006) found that emotional defensiveness, avoidant coping, and a high level of social support were risk factors for cancer. Reynolds et al. (2000) highlight the significance of low emotion expression and low perceived social support for the worse prognosis of patients with cancers. Furthermore, in Chinese studies on a group of patients with breast cancer (Wei et al., 2019), sense of coherence was negatively associated with Type C personality and depression, Type C personality was significantly positively associated with depression, and sense of coherence played a partial mediating role between Type C personality and depression, reducing the influence of Type C personality on depression.

In view of the diverse and not always consistent findings, an important research effort was the meta-analysis performed by McKenna et al. (1999), covering studies published from 1975 until 1996. It revealed that the factors significant in the development of cancer were: (a) denial/repression coping, (b) separation/loss experiences, (c) stressful life events, and (d) conflict-avoidant personality style (McKenna et al., 1999). These, however, are only four of the eight factors considered in the meta-analysis. Those that proved not to be significant were: increased anxiety/depression, childhood family environment (lack of support in childhood), difficulties with the expression of anger and resentment, and extraversion/introversion level. Another meta-analysis of 76 studies on depression and cancer mortality indicated that depression is associated with an increased risk of mortality in cancer patients and those who develop the disease (Pinquart and Duberstein, 2010). A meta-analysis conducted by Jokela et al. (2014) including six prospective cohort studies focused on the relationship between personality traits (extraversion, neuroticism, agreeableness, conscientiousness, and openness to experience) with cancer incidence and mortality as a result of cancer. The results indicated no association between any of the personality traits and the incidence of all cancers and any site-specific cancers (lung, colon, breast, prostate, skin, and leukemia/lymphoma) included in the analysis. None of the personality traits were also associated with cancer mortality.

It is worth noting that the role of personality and especially personality traits for occurrence and cancer courses are still heavily studied. In recent studies, on the one hand, de Mol et al. (2020) argue that personality traits are not associated with health-related quality of life and general quality of life, except for association between conscientiousness and physical health, in patients with advanced-stage lung cancer at the start of chemotherapy. However, on the other hand, several new studies suggest such relations; in particular, (a) neuroticism was strongly positively associated with emotional stress and mental health problems in oncological patients (Macía et al., 2020; Perry et al., 2020) and negatively associated with better health behaviors and health (Rochefort et al., 2018); (b) extraversion was positively associated with physical health, regardless of cancer diagnosis (Rochefort et al., 2018) and negatively associated with emotional stress and mental health problems in patients with cancer (Macía et al., 2020; Perry et al., 2020); when combined with two personality traits, a low level of neuroticism and a high level of extraversion were associated with better mental health (Macía et al., 2020); and (c) conscientiousness was weakly negatively associated with emotional stress in patients with cancer (Perry et al., 2020) and positively with better health behaviors and health (Rochefort et al., 2018).

Other characteristics of Type C personality can also be found in the literature, similar to the above to some extent, but also going beyond those already mentioned. Type C was supposed to characterize passive individuals, incapable of helping themselves, strongly focused on other people, unable to express their emotions, anger-repressing, helpless (Temoshok, 1987; Dolińska-Zygmunt, 2001a; Kurrass, 2004; Lehto et al., 2006; Ogden, 2007; Bozo et al., 2014; Lysaker et al., 2014), self-sacrificing (Kurrass, 2004), unable to manage their psychological behaviors, submissive, pathologically kind and agreeable, cooperative, excessively patient (Temoshok, 1987; Habibi et al., 2015), and showing excessive control of emotional features (Lysaker et al., 2014; Habibi et al., 2015); it has been attributed to individuals who have strong defense mechanisms resulting in the inability to verbalize and recognize negative emotions, who show secondary negative responses such as the sense of helplessness and uselessness, who lack self-control in stressful situations, and who are submissive to authorities (Temoshok, 1987).

The above characteristics of Type C personality show its high diversity. The problem of the consistency of these characteristics has not, essentially, been addressed at the theoretical level yet. The theoretical aim of the present article is to fill this gap.

### Problems Associated With Type C Personality

The literature devoted to Type C personality and its links with the occurrence of cancer contains many doubts and ambiguities. The major ones will be discussed below.

First, many research results that can be found in the literature do not confirm the significance of various components of Type C personality for the occurrence of cancer (cf. Schwarz and Geyer, 1984; Phillips et al., 2008; Archer et al., 2015). For example, Archer et al. (2015) did not confirm the link of chronic depressive symptoms with cancer occurrence. Phillips et al. (2008) found anxiety, depression, coping, and social support not to be significantly related to patients’ prognosis. In the study by Schwarz and Geyer (1984), demographic characteristics, serious stressful life events, and activity control were not significantly related to the occurrence of cancer.

Second, the description of Type C personality includes various contents with unclear structure, which may stem from the method used to construct the psychological variable referred to as Type C. This method consisted in Type C personality being distinguished based on the observation of the behaviors of patients suffering from cancer (Greer and Morris, 1975; Greer and Watson, 1985). Consequently, these behaviors constituted atheoretical indicators, observed in the behavior of patients diagnosed with this disease. Type C personality was analyzed in terms of its relations to other elements of personality structure to a small extent only, and its description relied on knowledge in the field of personality psychology to a very small degree. In the literature, it is possible to find different, sometimes, divergent or even contradictory characteristics attributed to Type C as the personality basis for cancer. For instance, Garssen and Goodkin (1999) underscored the significance of low social support for the development of cancer, whereas Lehto et al. (2006) highlighted the significance of high social support. Also, unclear and full of contradictions is the role of neuroticism and extraversion, which has been discussed above (Kissen and Eysenck, 1962; Eysenck, 1985, 1991; Blanchard and Abell, 2019).

Third, there are visible deficiencies in the acceptable operationalization of Type C. Even though the construct is interesting and attracts the attention of many researchers, it has not been properly operationalized and no sound measure has been developed to assess it. One of the best-known attempts at developing such measure is the questionnaire by Watson and Greer (1983); in view of the fact that the key element in Type C personality is usually considered to be emotional repression, these authors prepared a measure of emotional control (the Courtauld Emotional Control Scale, CECS), in which they distinguished three scales: Anger Control, Anxiety Control, and Depression Control. This questionnaire, however, does not measure Type C personality directly and can be seen as assessing only one element included in this construct.

Given the breadth of the construct, its special character, and the divergences concerning both the components distinguished in it and the results of research, one can conclude that the current measurement of Type C personality is inadequate and that a comprehensive operationalization of this construct is needed. Still, what is needed before the construction of the measure is a theoretical conceptualization and a precise definition of Type C, taking into account both the existing literature about it and the current knowledge about personality structure. The theoretical conceptualization of Type C is, therefore, a precondition of solving the third problem (i.e., the lack of comprehensive operationalization). It should be performed in such a way as to solve the second problem (i.e., conceptualization taking into account the current knowledge about personality structure), which, consequently, may give hope for a solution for the first problem (i.e., relation to cancer-proneness) in future research.

### Attempt to Solve the Problems Associated With Type C Personality: Conceptualization

We performed the postulated reconceptualization of Type C personality in four steps. In the first step, we compiled a list of all Type C contents and characteristics reported in the literature. In the second step, we combined those elements that were close to one another in terms of content, thus reducing the list of characteristics from Step 1. In the third step, we examined the structure of the groups distinguished and the possibilities of grouping the obtained elements into a smaller number of broader categories. In the fourth step, we linked the characteristics of Type C obtained in the previous steps with knowledge about personality structure. In particular, we looked at the characteristics of Type C from the point of view offered by the CPM (Strus et al., 2014; Strus and Cieciuch, 2017), which, on the one hand, integrates many models of temperament, personality, emotion, motivation, health, and well-being (Strus and Cieciuch, 2017), and which, on the other hand, can be seen as a tool for testing and refining constructs in other domains (see Cieciuch and Topolewska, 2017; Rogoza et al., 2019b).

#### The Reconstruction of the Contents of Type C (Steps 1–3)

In the first step, we identified 20 content units in the definitions of Type C present in the literature. These were: self-sacrifice, passiveness, calmness, peacefulness, strong focus on other people, submissiveness to others, submissiveness to authorities, pathological kindness, pathological agreeableness, excessive patience, cooperativeness, inability to help oneself, repression of negative emotions, strong defense mechanisms, inability to verbalize and identify the emotions experienced, sense of helplessness and uselessness, suppression of anger, excessive control of emotional symptoms, lack of self-control in stressful situations, and low awareness of the emotions experienced (Greer and Watson, 1985; Temoshok, 1987; Dolińska-Zygmunt, 2001a; Kurrass, 2004; Ogden, 2007; Bozo et al., 2014; Lysaker et al., 2014; Habibi et al., 2015).

In the second step, we grouped the above characteristics by combining semantically close contents. This resulted in six groups: (1) excessively high (pathological) kindness; (2) self-sacrifice, strong focus on other people, inability to help oneself; (3) peacefulness and excessive patience, pathological agreeableness and submissiveness; (4) repression of negative emotions, strong defense mechanisms, suppression of anger, excessive control of emotional symptoms, calmness; (5) low awareness of the emotions experienced, inability to verbalize and identify the emotions experienced; and (6) sense of helplessness and uselessness, lack of self-control in stressful situations, passiveness.

In the third step, we divided the contents from Step 2 into two domains: interpersonal and intrapersonal. The above presentation of contents in Step 2 was organized with a view to Step 3, which is why interpersonal contents are in Groups 1–3 and interpersonal contents are in Groups 4–6. The distinction between these two domains in Type C personality was inspired by the popular model of Type D personality structure (also referred to as distress personality) proposed by Denollet (2005).

The fourth step consisted in introducing an external frame of reference into the analysis of contents attributed to Type C from the perspective of personality psychology. The description of Type C had been developed as a description of behaviors or characteristics of individuals suffering from or prone to cancer. This process of construct development should be confronted with the current knowledge about personality structure. In personality psychology, there are many different models, which – as has been proposed for some time in the literature – can be integrated in the two-factor model of personality (DeYoung, 2015; Cieciuch and Strus, 2017), whose extension is the CPM (Strus et al., 2014; Strus and Cieciuch, 2017). This model offers a theoretical matrix that enables the synthesis and organization of various kinds of constructs, frequently also making it possible to conceptualize them more precisely. What can serve as an example is the integration of different perspectives on narcissism in CPM performed by Rogoza et al. (2019b), the integration of personality disorder categories performed by Zawadzki (2016, 2017), or the synthesis of various models in the area of identity formation performed by Cieciuch and Topolewska (2017), who applied the method itself rather than the CPM itself. Below, we will present the basic assumptions of CPM and then proceed to use the model to refine the theoretical definition of Type C and to further clarify its content aspects.

#### The Location of Type C Personality in the Circumplex of Personality Metatraits (Step 4)

**Figure 2:**
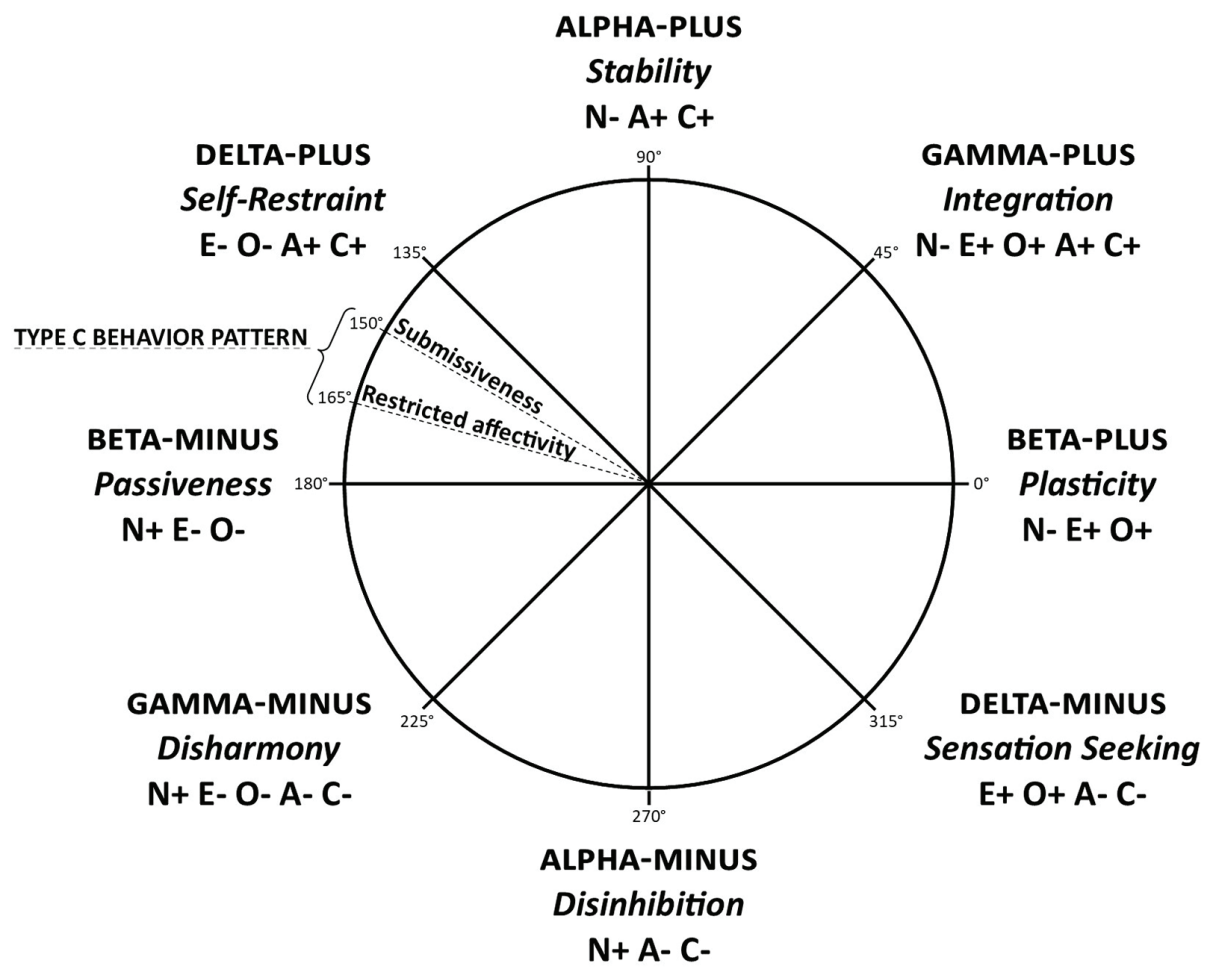
Graphic presentation of the CPM (modified by Strus et al., 2014). N, neuroticism; E, extraversion; O, openness to experience; A, agreeableness; C, conscientiousness; +, means the positive pole of the trait; −, means the negative pole of the trait.

The CPM model is an extension of the two-factor model of personality (Digman, 1997; DeYoung et al., 2002; Cieciuch and Strus, 2017), depicting the structure of personality at the level of two higher-order factors (metatraits): Alpha (Stability) and Beta (Plasticity). Dimensions of Alpha and Beta constitute a system of orthogonal coordinates, in which Strus et al. (2014) additionally distinguished the dimensions of Gamma (being a reinterpretation of the general factor of personality; Musek, 2007) and Delta (being a discovery that the logic of this model led to). In each metatrait, we distinguished two poles, whose psychological contents are not reducible to a pair of opposites. This resulted in an octantal structure. The current version of the model, modified after a series of studies (Strus and Cieciuch, 2017, 2019; Rogoza et al., 2019a), is presented in Figure 2.

CPM emerged from the tradition of research on the five-factor model of personality (McCrae and Costa, 2003), which is why Figure 2 presents the constellation of the Big Five traits for each metatrait. In turn, thanks to its circumplex structure, CPM creates a space in which the meaning of other constructs can be specified (see Rogoza et al., 2019a). If CPM is treated as an external system of coordinates synthesizing various models developed in the field of personality psychology, the question arises about the location of Type C within the circumplex. The scope of its contents presented above locates Type C between two metatraits: Delta-Plus/Self-Restraint (peacefulness, excessive patience, submissiveness, and adjustment in relations with people) and Beta-Minus/Passiveness (passiveness, helplessness and hopelessness, and low self-awareness). It seems that those personality characteristics that are situated in CPM between Delta-Plus and Beta-Minus – namely, high behavioral control, tendency to adjust oneself, conventionality, conformism, and submissiveness in interpersonal relations, repressed affectivity (both positive and negative) inhibition, apathy, and passiveness – correspond particularly strongly to the characteristics attributed to Type C personality (see Strus and Cieciuch, 2017). We considered this location of Type C personality in CPM as a broader integrating model of personality in order to theoretically clarify and refine Type C itself.

#### Definition of Type C

Considering the existing descriptions of Type C and its location in CPM, we propose the following definition and structure of Type C: Type C personality is composed of two facets: Submissiveness, corresponding to the interpersonal domain, and Restricted Affectivity, corresponding to the intrapersonal domain. Submissiveness and Restricted Affectivity have elements in common with both personality metratraits mentioned above, but Submissiveness is slightly closer to Delta-Plus/Self-Restraint, while Restricted Affectivity is closer to Beta-Minus/Passiveness. Submissiveness manifests itself in pathological agreeableness, compliance, kindness toward others, uncritical adjustment to them, dependence, excessive patience, peacefulness, inability to refuse, and even in excessive focus on other people and sacrificing oneself for them at the cost of one’s own needs. Restricted Affectivity manifests itself in the repression and suppression of negative emotions (particularly anger), low awareness of the emotions experienced, inability to identify, name, and express them, anhedonia, passiveness, and helplessness in the face of adversities.

### The Problem of the Present Study: Operationalization of Type C Personality

We subjected the conceptualization of Type C proposed above to a procedure of operationalization. In the first stage, we generated a pool of items measuring the components of Type C distinguished in the above definition (27 items for the Submissiveness scale and 17 items for the Restricted Affectivity scale). The aim of the study was to select items from the pool to be included in the final version of the questionnaire and to test its psychometric properties. We formulated the following hypotheses concerning the final version of the measure: internal consistency (measured with Cronbach’s alpha) is acceptable for both scales (Hypothesis 1). The measurement model in confirmatory factor analysis with two latent variables is well-fitted to the data set (Hypothesis 2). The measurement of Submissiveness and Restricted Affectivity is invariant at configural, metric, and scalar level across gender (Hypothesis 3). Both facets of Type C are located in the CPM between Delta-Plus/Self-Restraint and Beta-Minus/Passiveness; the predicted location of Submissiveness is closer to Delta-Plus, while the predicted location of Restricted Affectivity is closer to Beta-Minus, as shown in Figure 2 (Hypothesis 4).

## Materials and Methods

### Measures

#### Type C Personality

In the course of work on operationalizing the definition of Type C formulated above, our team generated 44 items: 27 items for the Submissiveness scale and 17 items for the Restricted Affectivity scale. We used a five-point Likert scale (from 1 – *completely untrue about me*, to 5 – *completely true about me*). This was the initial pool, from which, in the presented study, we selected the items for the final version of the measure.

We used four selection criteria. The first criterion consisted in removing those items that significantly positively correlated with well-being, as we decided that indicators of the potentially pathological Type C personality should not be positively related to mental health symptoms. The second criterion consisted in removing the items that did not differentiate respondents’ answers (low mean score and standard deviation). The items that remained after the selection performed according to the first two criteria were entered into the exploratory factor analysis for each of the two facets of Type C separately (which was the third criterion of selection). We removed the items with the lowest factor loadings. The fourth criterion was expert assessment of the contents of those items whose removal or retention was suggested by psychometric indicators. Decision concerning each item was based on theoretical reflection rather than made automatically.

#### Well-Being

To measure well-being, which was one of the item selection criteria for the measure of Type C, we used the following items: (1) “I often feel simply happy”; (2) “Little everyday things often give me joy”; (3) “If I could live my life again, I would change almost nothing”; and (4) “My natural mood can be called cheerfulness.” Answers were given on a five-point Likert scale, from 1 (*completely disagree*) to 5 (*completely agree*); Cronbach’s *α* was 0.70.

#### Personality Metatraits

To measure personality metatraits, we used the Circumplex of Personality Metatraits Questionnaire (CPM-Q-SF; Strus and Cieciuch, 2017). The questionnaire consists of 72 items describing a variety of human behaviors, feelings, thoughts, and attitudes. It measures the eight metatraits distinguished in CPM: Alpha-Plus/Stability, Alpha-Minus/Disinhibition, Beta-Plus/Plasticity, Beta-Minus/Passiveness, Gamma-Plus/Integration, Gamma-Minus/Disharmony, Delta-Plus/Self-Restraint, and Delta-Minus/Sensation Seeking. Answers were given on a five-point Likert scale from 1 (*completely disagree*) to 5 (*completely agree*). Cronbach’s *α* of the scales ranged from 0.72 (Alpha-Plus/Stability) to 0.85 (Gamma-Minus/Disharmony).

### Participants

The participants in the study were 232 individuals aged 18–70 (*M* = 29.35, *SD* = 8.93). Men (*n* = 126) constituted 54.3% of the sample. The participants completed the paper-and-pencil version of the set of measures; their anonymity was fully ensured. When collecting the data, we were aided by psychology students, who recruited participants among their friends, acquaintances, and distant relatives. The inclusion criteria was age (18 years or more) and approximate equal number of men and women.

## Results

We removed seven items after applying Criterion 1 of item selection (see description in the Measures section), and we eliminated two further items after applying Criterion 2. By applying Criteria 3 and 4, we selected 10 items for each scale, which are presented in Table 1.

**Table 1:** Factor loadings in the confirmatory factor analysis of the questionnaire measuring Type C.

	Submissiveness	Restricted affectivity
I believe it is my obligation to put other people’s needs above my own.	0.77	
In conflict situations, I always yield and submit to others.	0.70	
I can accept anything, even if it does not suit me.	0.66	
I cannot say “no” to others.	0.61	
Even if something does not suit me, I do not say it.	0.63	
I am ready to give up my plans for the good of others at any time.	0.61	
I always agree with the people important to me, because I know they are right.	0.61	
I am absolutely determined to respond to the needs of others, regardless of how I feel.	0.60	
Regardless of the situation, I wait for my turn.	0.59	
I do what I can to avoid conflicts with others.^*^	0.55	
All my life I have not expressed my strong emotions.		0.66
When something goes wrong, I simply come to terms with it.		0.60
To be honest, I never actually feel anger.		0.57
In difficult situation, I usually submit to the course of events.		0.56
I never actually reflect on what I feel.		0.57
No one can really make me upset.		0.53
When life is not going my way, I accept that this is how it has to be.		0.52
As a matter of fact, nothing can make me lose my temper.		0.47
When something bad happens to me, I do not think about it at all.		0.43
No matter how bad I feel, I do not show it.^**^		0.39

* Proposed modification for use in future studies: I do what I can to avoid conflicts with others at all costs.

** Proposed modification for use in future studies: No matter how I feel, I do not show it.

Internal consistency (Cronbach’s alpha) was 0.85 for Submissiveness and 0.79 for Restricted Affectivity, which can be regarded as acceptable values, confirming Hypothesis 1.

In categorical confirmatory factor analysis (after the inclusion of seven error correlations between items similar in meaning), the measurement model had the following measurement fit: chi^2^ = 353.6, *df* = 162, RMSEA = 0.071 (0.061–0.082), CFI = 0.927, WRMR = 1.06. The correlation between the latent variables was 0.66. With the obtained fit indices, Hypothesis 2 can be considered as confirmed.

In order to test for measurement invariance (Hypothesis 3) across gender, we ran a multigroup confirmatory factor analysis. For technical reasons, in six cases, one answer was randomly changed to the neighboring value (e.g., answer 4 was changed to 5) in order to have all answers for all items in all groups, which is a requirement for categorical measurement invariance. We obtained the following model fit indices (a) at the configural level: chi^2^ = 524.9, *df* = 324, CFI = 0.927, RMSEA = 0.073 (0.061–0.084); (b) at the metric level: chi^2^ = 552.7, *df* = 342, CFI = 0.923; RMSEA = 0.073 (0.061–0.084); and (c) at the scalar level: chi^2^ = 622.0, *df* = 400, CFI = 0.919, RMSEA = 0.069 (0.058–0.080). According to commonly used criteria (Cieciuch and Davidov, 2015), one can conclude that configural, metric, and scalar measurement invariance across gender is established. This means that both facets of Type C have the same meaning (metric invariance) and are measured in the same way (scalar invariance) across gender.

**Figure 3:**
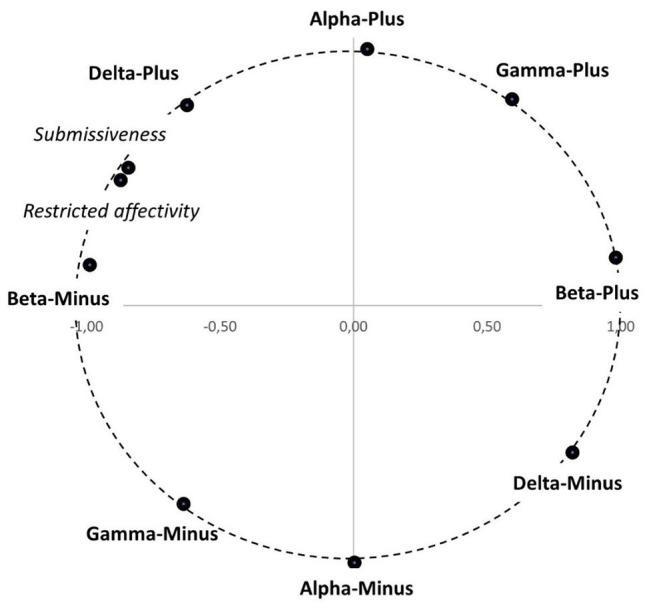
Factor loading plots for CPM metatraits and facets of Type C from the rotated matrix with row normalization.

We tested Hypothesis 4 using the procedure of orthogonal Procrustes rotation (Schönemann, 1966), which Strus and Cieciuch (2017) had applied when testing hypotheses concerning the location of variables in CPM and which is recommended by Rogoza et al. (2019a) for analyzing circumplex models. The aim of the analyses was to compare the theoretical location of the facets of Type C (Submissiveness at 150 degree and Restricted Affectivity at 165 degree) with their actual empirical location. The theoretical angular location is converted in accordance with trigonometric functions into coordinates (*target matrix*), and the empirical location is the factor loadings of the analyzed variables in the two-factor solution (*obtained matrix*). Procrustes rotation rotates the empirically obtained results to the theoretically expected ones without changing the results. There are two types of Procrustes rotations: with or without row normalization. Both of them are presented in Table 2, while Figure 3 presents the results with row normalization. In the rotation with row normalization, empirical loadings are transformed to the same unit-metric space as the target matrix (coordinates of 1 and +1). Factor loadings can be converted into angles, in accordance with trigonometric functions, and the angles can be compared with the theoretically expected ones. The degree of similarity for the entire matrix and for each variable separately is expressed as the congruence coefficient. It is assumed that a coefficient value >0.90 indicates high congruence and a value >0.95 indicates very high congruence (Barrett, 1986; Lorenzo-Seva and ten Berge, 2006). We also report *R*^2^, which is a measure of explained variance in a given variable explained by other variables included in the model.

**Table 2:** Target and obtained matrices for Circumplex of Personality Metatraits (CPM) and the two facets of Type C.

	ϴ^T^	Target matrix		Obtained matrix row		Obtained matrix normalized		*R*^2^	Congr.	ϴ^E^
		F1	F2	F1	F2	F1	F2			
Delta-Plus	135	0.71	−0.71	0.64	−0.50	0.78	−0.62	0.65	0.99	128.61
Alpha-Plus	90	1.00	0.00	0.82	0.06	1.00	0.05	0.68	1.00	86.99
Gamma-Plus	45	0.71	0.71	0.68	0.52	0.80	0.59	0.74	0.99	53.58
Beta-Plus	0	0.00	1.00	0.14	0.82	0.19	0.98	0.69	0.98	10.83
Delta-Minus	315	−0.71	0.71	−0.46	0.63	−0.57	0.82	0.61	0.99	325.11
Alpha-Minus	270	−1.00	0.00	−0.89	−0.01	−1.00	0.00	0.79	1.00	270.09
Gamma-Minus	225	−0.71	−0.71	−0.60	−0.51	−0.77	−0.64	0.63	1.00	230.46
Beta-Minus	180	0.00	−1.00	0.15	−0.86	0.16	−0.99	0.77	0.98	170.89
Submissiveness	150	0.50	−0.87	0.23	−0.34	0.54	−0.84	0.17	1.00	147.52
Restricted affectivity	165	0.26	−0.97	0.21	−0.35	0.49	−0.87	0.17	0.97	150.76
Factor/overall congruence				0.98	0.96				0.99	

Target matrix is based on the hypothesized circumplex structure shown in Figure 2. $\theta^{T}$, angle theoretically predicted; $\theta^{E}$, angle empirically obtained (angles in degrees); F1 and F2, factors; $R^{{}^{2}}$, explained variance coefficients; Congr., congruence coefficients.

Table 2 presents target and obtained matrices for CPM and the two facets of Type C, followed by congruence coefficient, *R*^2^, and empirically obtained angle.

As expected, the two facets of Type C were located close to each other, in the space between Delta-Plus and Beta-Minus, in places theoretically predicted, as evidenced by the very high congruence coefficients. The obtained results make it reasonable to consider Hypothesis 3 confirmed, but two issues should be pointed out. First, despite the high congruence coefficients, the two facets of Type C were located very close to each other – somewhat closer than expected. What we expected was that they would be located at 15-degree intervals between Beta-Minus and Delta-Plus, whereas the empirical results actually placed both facets halfway between Beta-Minus and Delta-Plus. Second, *R*^2^ is relatively low, which means that the metatraits distinguished in the CPM do not explain a particularly high proportion of the variance in Type C. This result means that Type C is not reducible to personality metatraits, even though it is quite precisely located in the space defined by them.

## Discussion

In research aimed at finding the personality determinants of somatic diseases, Type C personality has been proposed and treated as a significant predictor of the incidence of cancer. The analysis of the available literature on Type C has revealed a number of characteristics postulated as elements of Type C, which were not always internally consistent and did not form a precisely defined whole. This might have been one of the causes behind the divergence, reported in the literature, in research results concerning the significance of Type C personality in predicting cancers. The relatively blurred meaning of this construct and the wide scope of characteristics included in it certainly did not facilitate its operationalization, which might have been an argument in favor of measuring only selected components rather than attempting to capture Type C as a whole.

We see our research as the first step toward clarify the theorizing and results on Type C personality. The key element of our approach was the literature-based attempt to organize the components of Type C, which led to its reconceptualization. It was on the theoretical plane, thus built that we proposed a comprehensive operationalization of Type C. The measure we developed was preliminarily found to have acceptable psychometric properties (internal consistency, factorial validity, and external validity).

Of course, what remains an unsolved problem is the significance of Type C for cancer (its appearance, development, and treatment prognosis). This problem should be addressed by future studies, or even by entire research programs. However, we propose a modification in the overall approach to this kind of research. The approach, so far, has been to find atheoretical personality indicators of cancer by looking for the typical characteristics of people suffering from it. As meta-analyses have shown, this approach did not lead to conclusive results (McKenna et al., 1999). Moreover, in some studies, no association between any of the personality traits and cancer and cancer mortality was found (Jokela et al., 2014). One of the causes might have been the primacy of direct observation over the strength of a good theoretical model. This primacy resulted in little reflection being undertaken on the theoretical meaning of Type C construct, on its internal consistency and structure, and on its location in broader models of personality structure. The approach we have proposed overcomes these weaknesses and uses the CPM (Strus et al., 2014) as the theoretical personality context and a point of reference. It should be stressed that CPM does not absorb other constructs in such a way as to make them redundant. On the contrary, it turned out that Type C was irreducible to metatraits but it was worth applying the logic of CPM in its description and structure. This resulted in an internally consistent construct that has a well-defined structure and is clearly related to the broader model of personality.

Perhaps, it will turn out that our conceptualization and operationalization of Type C will not solve problems associated with the personality determinants of cancer but it seems that this kind of personality structure attempt is necessary. It can be said that this means giving Type C a chance as a reformed and reconceptualized theoretical construct in its role of a predictor of psychosomatic problems. Time will tell if this proves to be a chance of success or failure. It seems, however, that without this chance Type C personality would be slowly receding into the history of psychology.

## Data Availability Statement

The raw data supporting the conclusions of this article will be made available by the authors, without undue reservation.

## Ethics Statement

The studies involving human participants were reviewed and approved by Commission of Ethics and Bioethics at the Cardinal Stefan Wyszyński University in Warsaw. Written informed consent for participation was not required for this study in accordance with the national legislation and the institutional requirements.

## Author Contributions

KR and AT prepared the first set of items to measure Type C. WS, KR, AT, and JC prepared the reconceptualization of Type C. KR, WS, and JC prepared the final set of items, designed the study, and run the analysis. KR in collaboration with JC and WS prepared the first version of the paper. JC revised the paper. All authors contributed to the article and approved the submitted version.

### Conflict of Interest

The authors declare that the research was conducted in the absence of any commercial or financial relationships that could be construed as a potential conflict of interest.
